# The evolution of crocodilian nesting ecology and behavior

**DOI:** 10.1002/ece3.5859

**Published:** 2019-12-12

**Authors:** Christopher M. Murray, Brian I. Crother, J. Sean Doody

**Affiliations:** ^1^ Department of Biology Tennessee Technological University Cookeville TN USA; ^2^ Department of Biology Southeastern Louisiana University Hammond LA USA; ^3^ Department of Biological Sciences University of South Florida ‐ St. Petersburg St. Petersburg FL USA

**Keywords:** character evolution, crocodilians, eggs, nesting, phylogeny, reproduction

## Abstract

Crocodilians comprise an ancient and successful lineage of archosaurs that repeatedly raises questions on how they survived a mass extinction and remained relatively unchanged for ~100 million years. Was their success due to the change‐resistant retention of a specific set of traits over time (phylogenetic conservatism) or due to flexible, generalist capabilities (e.g., catholic diets, phenotypic plasticity in behavior), or some combination of these? We examined the evolution of reproductive ecology and behavior of crocodilians within a phylogenetic perspective, using 14 traits for all 24 species to determine whether these traits were phylogenetically constrained versus (ecologically) convergent. Our analysis revealed that the ancestral crocodilian was a mound nester that exhibited both nest attendance and defense. Nesting mode exhibited 4–5 transformations from mound to hole nesting, a convergence of which habitat may have been a driving factor. Hole nesters were more likely to nest communally, but this association may be biased by scale. Although there were exceptions, mound nesters typically nested during the wet season and hole nesters during the dry season; this trait was relatively conserved, however. About two‐thirds of species timed their *nesting* with the wet season, while the other third timed their *hatching* with the onset of the wet season. Nest attendance and defense were nearly ubiquitous and thus exhibited phylogenetic conservatism, but attendance lodging was diverse among species, showing multiple reversals between water and burrows. Collectively, our analysis reveals that reproductive trait evolution in crocodilians reflects phylogenetic constraint (nest attendance, nest defense), ecological convergence (seasonal timing of nesting, nest attendance lodging), or both (mode of nesting). Some traits (e.g., communal nesting and mode of nesting) were autocorrelated. Our analysis provides a framework for addressing hypotheses raised for why there has been trait convergence in reproductive ecology and behavior in crocodilians and why some traits remained phylogenetically conserved.

## Graphical abstract

Our analysis reveals that reproductive trait evolution in crocodilians reflects phylogenetic constraint (nest attendance, nest defense), ecological convergence (seasonal timing of nesting, nest attendance lodging), or both (mode of nesting). Our analysis provides a framework for addressing hypotheses raised for why there has been trait convergence in reproductive ecology and behavior in crocodilians and why some traits remained phylogenetically conserved.

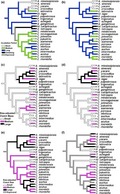

## 1 INTRODUCTION

A modern goal of evolutionary biologists is to bridge the conceptual divide between microevolution and macroevolution (Bock, 1970; Hansen & Martins, 1996; Reznick & Ricklefs, 2009; Gavrilets & Losos, 2009; Rabosky, 2013). Although this goal is fraught with challenges, the stage may be set for re‐evaluation; according to Erwin (2000) and Reznick and Ricklefs (2009), it is the integration of information from the fossil record, the population and evolutionary dynamics of extant organisms, and phylogenetics that will provide the ultimate test of the bridge between microevolution and macroevolution (but see Tobias et al., 2014). Regardless, macroevolution can inform microevolution: Information about the historical patterns of diversification of lineages can now be mined from molecular phylogenies, potentially shedding light on the underlying causes of these patterns (Reznick & Ricklefs, 2009).

If we define success as the longevity of a lineage, the clade that comprises the living crocodilians must rank as highly successful. The Crocodylia as we know them are ~100 million years old and carry the legacy of their archosaur cousins, the dinosaurs, as the largest reptiles on earth. They present modest species richness (25 species), generalist habits, and wide‐ranging, amphibious capabilities. In any case, their success raises questions on what facets of their biology might explain their longevity as a group—for example, how did the crocodilian lineage survive the K‐T extinction when their cousins the dinosaurs did not? Was it due to the change‐resistant retention of a specific set of natural history traits over geological time (phylogenetic constraint) or due to their generalist capabilities (e.g., catholic diets, phenotypic plasticity in behavior), or some combination of these?

The most recent review of crocodilian biology seems to argue for the former; Grigg and Kirshner (2015) repeatedly highlight similarities in the physiology, ecology, and behavior within the group. For example, they note that differences in the ecology and behavior between the Crocodylidae and Alligatoridae are “surprisingly few,” given their existence for ~100 million years. Indeed, all living crocodilians are aquatic generalist (strict) carnivores, engage in mating vocalizations, possess temperature‐dependent sex determination, and exhibit considerable parental care to their eggs and neonates (reviewed in Webb & Manolis, 1989, and in Grigg & Kirshner, 2015).

Despite these similarities and others involving form and function, the reproductive ecology and behavior of crocodilians exhibit intriguing diversity. A major example is mode of nesting: Some species excavate a hole nest in the substrate, while other species build a mound nest out of surrounding vegetation (Campbell, 1972; Greer, 1970; Neill, 1971). This example represents one “striking dichotomy” (Grigg & Kirshner, 2015) that has been discussed but remains unresolved (Bayani, Trivedi, & Suresh, 2011; Brazaitis & Watanabe, 2011; Greer, 1970; Grigg, Thompson, Beard, & Harlow, 2010; Thorbjarnarson, 1996). Further, when considering long‐lived iteroparous species, parental behavior, nesting habitat, and size‐based reproductive output vary greatly among species. Marked variation in egg mass, clutch size, and clutch frequency with respect to maternal size among crocodilian species (reviewed in Thorbjarnarson, 1996) lends to evolutionary lability in behavioral and reproductive traits as an explanation for their historical success. According to Grigg and Kirshner (2015), “no explanation has yet emerged that accounts for this striking dichotomy in behavior.”

The opportunity for understanding the evolution of reproductive ecology and behavior has arguably never been better. First, a recent robust phylogenetic analysis (Oaks, 2011) provides a framework for tracking reproductive trait evolution in crocodilians. Second, there has been a major surge in new information on the biology of crocodilians in recent years and decades (Grigg & Kirshner, 2015). For example, of the ~1,100 publications cited in Grigg and Kirshner (2015), 90% have been published since 1970 and 45% of them since 2000. Naturally, reviews and syntheses have lagged behind, including those on reproductive ecology and behavior (but see Magnusson, Lima, & Sampaio, 1985; Thorbjarnarson, 1996, for exceptions). Data on reproductive ecology are now available for all 24 species; however, and collectively, these prospects make crocodilians a good choice for studying reproductive trait evolution.

Here, we examine the evolution of reproductive ecology and behavior of crocodilians from a phylogenetic perspective, using 14 traits for all 24 species. Specifically, we pose the following questions: (a) Which traits associated with crocodilian reproductive ecology and behavior are phylogenetically constrained versus (ecologically) convergent? (b) How do these findings accord with previous hypotheses on reproductive ecology and behavior in crocodilians? (c) What were the reproductive traits (character states) of the hypothetical ancestor(s) of the Crocodylia? We discuss interrelationships between different traits within the context of the ecology and behavior of crocodilians and pose further questions for investigation.

## 2 METHODS

The evolutionary relationships of crocodilians have been plagued by incongruence among studies (Brochu, 2003; Poe, 1996). Aside from questions pertaining to the timing of diversification, there has been no consensus on the placement of the false or Sundha gharial (*Tomistoma *sp.) as sister to *Gavialis* or to the family Crocodylidae (Gatesy, Amato, Norell, DeSalle, & Hayashi, 2003). However, a recent robust analysis (Oaks, 2011) yields a monophyletic Gavialidae (comprised of *Tomistoma* and *Gavialis*) as sister to Crocodylidae, and provides an opportunity to determine the taxonomic distribution, and thus reproductive trait evolution, in crocodilians.

Character states for 14 characters were collected for all 24 species from published literature (Table 1). The suite of characters chosen attempts to characterize all the variable modes of behavioral and ecological investment allocated to reproduction among crocodilians. The character states overwhelmingly found in the literature were assigned to that taxon. For example, the character state for nest defense in the American crocodile (*Crocodylus acutus*) was assigned as “absent” (obtained from Charruau & Hénaut, 2012) despite a note of present defense in mothers at the Smithsonian Tropical Research Institute in Panama (Paez & Bock, 1988). Continuous variables (size‐adjusted relative clutch mass, clutch size, and incubation duration) were taken from Thorbjarnarson (1996) and were transformed to categorical using the gap weighting method described by Thiele (1993). Character states were optimized onto the crocodilian phylogeny of Oaks (2011) using MacClade (version 4.08; Maddison & Maddison, 2005) under a most parsimonious reconstruction framework using delayed transformation (DELTRAN), an algorithm favoring bottom‐up character state assignments favoring parallelisms, and accelerated transformation (ACCTRAN), an algorithm favoring top‐down character state assignments favoring reversals. We eschewed the use of probabilistic methods for inferring ancestral states because of the absence of branch lengths and because of general problems associated with models of ancestral state reconstruction (e.g., Ekman, Andersen, & Wedin, 2008).

**Table 1:** Characters and cited published literature sources

Species	Character	Literature source(s)
*Alligator mississippiensis*	Nest site (**mound**, hole, termite)	Joanen and McNease (1980)
	Communal nesting (yes, **no**)	No report of occurrence
	Nest defense (**yes**, no)	Joanen and McNease (1980)
	Nest attendance (**yes**, no)	Joanen and McNease (1980)
	Hatchling attendance (**yes**, no)	Joanen (1969)
	Attendance lodging (**water, burrow**, wallow)	Joanen (1969)
	Hatching stimulus (**signal calling**, vibration)	Joanen (1969)
	Nesting season (**wet**, dry)	Joanen and McNease (1980)
	Clutch frequency (**1**, 2)	Joanen and McNease (1980)
	Clutch size (small, medium, **large**)	Thorbjarnarson (1996)
	Clutch mass (**small**, medium, large)	Thorbjarnarson (1996)
	Mating vocals (none, roar, **bellow**, buzz, moan)	Vliet (1987)
	Wet season hatching (yes, **no**)	Joanen and McNease (1980)
	Incubation duration (**short**, medium, long, extralong)	Thorbjarnarson (1996)
*Alligator sinensis*	Nest site (**mound**, hole, termite)	Thorbjarnarson, Wang, and He (2001)
	Communal nesting (yes, **no**)	No report of occurrence
	Nest defense (**yes**, no)	Platt, Li, He, Wang, and Shunqing (2016)
	Nest attendance (yes, no)	No data
	Hatchling attendance (**yes**, no)	Thorbjarnarson et al. (2001)
	Attendance lodging (water, burrow, wallow)	No data
	Hatching stimulus (**signal calling**, vibration)	Thorbjarnarson et al. (2001)
	Nesting season (**wet**, dry)	Thorbjarnarson et al. (2001)
	Clutch frequency (**1**, 2)	Thorbjarnarson et al. (2001)
	Clutch size (small, **medium**, large)	Thorbjarnarson (1996)
	Clutch mass (small, **medium**, large)	Thorbjarnarson (1996)
	Mating vocals (none, roar, **bellow**, buzz, moan)	Thorbjarnarson et al. (2001)
	Wet season hatching (yes, **no**)	Thorbjarnarson et al. (2001)
	Incubation duration (**short**, medium, long, extralong)	Thorbjarnarson (1996)
*Caiman latirostris*	Nest site (**mound**, hole, termite)	Poletta et al. (2011)
	Communal nesting (**yes**, no)	Larriera (2002)
	Nest defense (**yes**, no)	Larriera and Piña (2000)
	Nest attendance (**yes**, no)	Larriera and Piña (2000)
	Hatchling attendance (**yes**, no)	Larriera and Piña (2000)
	Attendance lodging (water, burrow, wallow)	No data
	Hatching stimulus (**signal calling**, vibration)	Staton and Dixon (1977)
	Nesting season (**wet**, dry)	Simoncini, Marcó, Portelinha, and Piña (2016)
	Clutch frequency (**1**, 2)	Simoncini et al. (2016)
	Clutch size (small, medium, **large**)	Thorbjarnarson (1996)
	Clutch mass (small, medium, **large**)	Thorbjarnarson (1996)
	Mating vocals (none, roar, **bellow**, buzz, moan)	Gorzula and Seijas (2015)
	Wet season hatching (yes, **no**)	Simoncini et al. (2016)
	Incubation duration (short, **medium**, long, extralong)	Thorbjarnarson (1996)
*Caiman crocodilus*	Nest site (**mound**, hole, termite)	Allsteadt (1994)
	Communal nesting (yes, **no**)	No report of occurrence
	Nest defense (**yes**, no)	Allsteadt (1994)
	Nest attendance (**yes**, no)	Allsteadt (1994)
	Hatchling attendance (**yes**, no)	Allsteadt (1994)
	Attendance lodging (**water**, burrow, wallow)	Allsteadt (1994)
	Hatching stimulus (signal calling, vibration)	No data
	Nesting season (**wet**, dry)	Allsteadt (1994)
	Clutch frequency (**1**, 2)	Allsteadt (1994)
	Clutch size (small, **medium**, large)	Thorbjarnarson (1996)
	Clutch mass (small, medium, **large**)	Thorbjarnarson (1996)
	Mating vocals (none, **roar**, bellow, buzz, moan)	Dinets (2013)
	Wet season hatching (yes, **no**)	Allsteadt (1994)
	Incubation duration (**short**, medium, long, extralong)	Thorbjarnarson (1996)
*Caiman yacare*	Nest site (**mound**, hole, termite)	Cintra (1988)
	Communal nesting (yes, **no**)	No report of occurrence
	Nest defense (**yes**, no)	Cintra (1988)
	Nest attendance (**yes**, no)	Cintra (1988)
	Hatchling attendance (**yes**, no)	Cintra (1988)
	Attendance lodging (**water**, burrow, wallow)	Cintra (1988)
	Hatching stimulus (signal calling, vibration)	No data
	Nesting season (**wet**, dry)	Cintra (1988)
	Clutch frequency (**1**, 2)	Cintra (1988)
	Clutch size (small, **medium**, large)	Thorbjarnarson (1996)
	Clutch mass (small, **medium**, large)	Thorbjarnarson (1996)
	Mating vocals (none, **roar**, bellow, buzz, moan)	Dinets (2013)
	Wet season hatching (yes, **no**)	Cintra (1988)
	Incubation duration (**short**, medium, long, extralong)	Thorbjarnarson (1996)
*Melanosuchus niger*	Nest site (**mound**, hole, termite)	VillamarÍn‐Jurado and Suárez (2007)
	Communal nesting (yes, **no**)	No report of occurrence
	Nest defense (**yes**, no)	VillamarÍn‐Jurado and Suárez (2007)
	Nest attendance (**yes**, no)	Herron, Emmons, and Cadle (1990)
	Hatchling attendance (**yes**, no)	VillamarÍn‐Jurado and Suárez (2007)
	Attendance lodging (**water**, burrow, wallow)	VillamarÍn‐Jurado and Suárez (2007)
	Hatching stimulus (**signal calling**, vibration)	VillamarÍn‐Jurado and Suárez (2007)
	Nesting season (**wet**, dry)	VillamarÍn‐Jurado and Suárez (2007)
	Clutch frequency (**1**, 2)	F. Villamarin (personal communication)
	Clutch size (small, medium, **large**)	Thorbjarnarson (1996)
	Clutch mass (small, **medium**, large)	Thorbjarnarson (1996)
	Mating vocals (none, **roar**, bellow, buzz, moan)	Dinets (2013)
	Wet season hatching (yes, **no**)	VillamarÍn‐Jurado and Suárez (2007)
	Incubation duration (short, medium, **long**, extralong)	Thorbjarnarson (1996)
*Paleosuchus trigonatus*	Nest site (**mound**, hole, termite)	Magnusson et al. (1985)
	Communal nesting (yes, **no**)	No report of occurrence
	Nest defense (**yes**, no)	Campos, Muniz, Desbiez, and Magnusson (2016)
	Nest attendance (**yes**, no)	Campos et al. (2016)
	Hatchling attendance (yes, no)	No data
	Attendance lodging (water, burrow, **wallow**)	Campos et al. (2016)
	Hatching stimulus (signal calling, vibration)	No data
	Nesting season (wet, **dry**)	Magnusson et al. (1985)
	Clutch frequency (**1**, 2)	Magnusson et al. (1985)
	Clutch size (**small**, medium, large)	Thorbjarnarson (1996)
	Clutch mass (small, medium, **large**)	Thorbjarnarson (1996)
	Mating vocals (none, **roar**, bellow, buzz, moan)	Dinets (2013)
	Wet season hatching (**yes**, no)	Magnusson et al. (1985)
	Incubation duration (short, medium, long, **extralong**)	Thorbjarnarson (1996)
*Paleosuchus palpebrosus*	Nest site (**mound**, hole, termite)	Magnusson and Campos (2010)
	Communal nesting (yes, **no**)	No report of occurrence
	Nest defense (yes, no)	No data
	Nest attendance (**yes**, no)	Magnusson and Campos (2010)
	Hatchling attendance (**yes**, no)	Campos, Sanaiotti, Muniz, Farias, and Magnusson (2012)
	Attendance lodging (water, **burrow**, wallow)	Campos et al. (2012)
	Hatching stimulus (signal calling, vibration)	No data
	Nesting season (**wet**, dry)	Magnusson and Campos (2010)
	Clutch frequency (**1**, 2)	Campos et al. (2012)
	Clutch size (**small**, medium, large)	Thorbjarnarson (1996)
	Clutch mass (small, medium, **large**)	Thorbjarnarson (1996)
	Mating vocals (none, **roar**, bellow, buzz, moan)	Dinets (2013)
	Wet season hatching (yes, **no**)	Magnusson and Campos (2010)
	Incubation duration (short, medium, **long**, extralong)	Thorbjarnarson (1996)
*Crocodylus acutus*	Nest site (mound, **hole**, termite)	Murray, Easter, Padilla, Marin, and Guyer (2016)
	Communal nesting (**yes**, no)	Murray (personal communication)
	Nest defense (yes, **no**)	Murray et al. (2016)
	Nest attendance (**yes**, no)	Murray et al. (2016)
	Hatchling attendance (yes, **no**)	Murray et al. (2016)
	Attendance lodging (water, **burrow**, wallow)	Charruau and Hénaut (2012)
	Hatching stimulus (**signal calling**, vibration)	Garrick and Lang (1977)
	Nesting season (wet, **dry**)	Murray et al. (2016)
	Clutch frequency (**1**, 2)	Murray et al. (2016)
	Clutch size (small, medium, **large**)	Thorbjarnarson (1996)
	Clutch mass (**small**, medium, large)	Thorbjarnarson (1996)
	Mating vocals (none, **roar**, bellow, buzz, moan)	Dinets (2013)
	Wet season hatching (**yes**, no)	Murray et al. (2016)
	Incubation duration (short, medium, **long**, extralong)	Thorbjarnarson (1996)
*Crocodylus intermedius*	Nest site (mound, **hole**, termite)	Thorbjarnarson and Hernández (1993)
	Communal nesting (yes, **no**)	No report of occurrence
	Nest defense (**yes**, no)	Thorbjarnarson and Hernández (1993)
	Nest attendance (**yes**, no)	Thorbjarnarson and Hernández (1993)
	Hatchling attendance (**yes**, no)	Thorbjarnarson and Hernández (1993)
	Attendance lodging (water, burrow, wallow)	No data
	Hatching stimulus (**signal calling**, vibration)	Thorbjarnarson and Hernández (1993)
	Nesting season (wet, **dry**)	Thorbjarnarson and Hernández (1993)
	Clutch frequency (**1**, 2)	Thorbjarnarson and Hernández (1993)
	Clutch size (small, medium, **large**)	Thorbjarnarson (1996)
	Clutch mass (**small**, medium, large)	Thorbjarnarson (1996)
	Mating vocals (none, **roar**, bellow, buzz, moan)	Dinets (2013)
	Wet season hatching (**yes**, no)	Thorbjarnarson and Hernández (1993)
	Incubation duration (short, **medium**, long, extralong)	Thorbjarnarson (1996)
*Crocodylus morletii*	Nest site (**mound**, hole, termite)	Platt, Sigler, and Rainwater (2010)
	Communal nesting (yes, **no**)	No report of occurrence
	Nest defense (**yes**, no)	Platt et al. (2010)
	Nest attendance (**yes**, no)	Platt et al. (2010)
	Hatchling attendance (**yes**, no)	Platt et al. (2010)
	Attendance lodging (**water**, **burrow**, wallow)	Platt (2000)
	Hatching stimulus (**signal calling**, vibration)	Platt et al. (2010)
	Nesting season (**wet**, dry)	Platt et al. (2010)
	Clutch frequency (**1**, 2)	Platt et al. (2010)
	Clutch size (small, **medium**, large)	Thorbjarnarson (1996)
	Clutch mass (small, **medium**, large)	Thorbjarnarson (1996)
	Mating vocals (none, **roar**, bellow, buzz, moan)	Dinets (2013)
	Wet season hatching (**yes**, no)	Platt et al. (2010)
	Incubation duration (short, **medium**, long, extralong)	Thorbjarnarson (1996)
*Crocodylus rhombifer*	Nest site (**mound, hole**, termite)	Targarona et al. (2010)
	Communal nesting (yes, **no**)	No report of occurrence
	Nest defense (**yes**, no)	Lahrsson and Wihman (1989)
	Nest attendance (**yes**, no)	Lahrsson and Wihman (1989)
	Hatchling attendance (yes, no)	No data
	Attendance lodging (**water**, burrow, wallow)	Targarona et al. (2010)
	Hatching stimulus (signal calling, vibration)	No data
	Nesting season (**wet**, dry)	Targarona et al. (2010)
	Clutch frequency (**1**, 2)	Lahrsson and Wihman (1989)
	Clutch size (small, **medium**, large)	Thorbjarnarson (1996)
	Clutch mass (**small**, medium, large)	Thorbjarnarson (1996)
	Mating vocals (none, **roar**, bellow, buzz, moan)	Lahrsson and Wihman (1989)
	Wet season hatching (**yes**, no)	Ramos Targarona, Soberón, Tabet, and Thorbjarnarson (2010)
	Incubation duration (short, **medium**, long, extralong)	Thorbjarnarson (1996)
*Crocodylus niloticus*	Nest site (mound, **hole**, termite)	Modha (1967)
	Communal nesting (**yes**, no)	Swanepoel, Ferguson, and Perrin (2000)
	Nest defense (**yes**, no)	Pooley (1977)
	Nest attendance (**yes**, no)	Kofron (1989)
	Hatchling attendance (**yes**, no)	Modha (1967)
	Attendance lodging (water, burrow, wallow)	No data
	Hatching stimulus (**signal calling**, vibration)	Pooley (1977)
	Nesting season (wet, **dry**)	Kofron (1989)
	Clutch frequency (**1**, 2)	No data
	Clutch size (small, medium, **large**)	Thorbjarnarson (1996)
	Clutch mass (**small**, medium, large)	Thorbjarnarson (1996)
	Mating vocals (none, **roar**, bellow, buzz, moan)	Dinets (2013)
	Wet season hatching (yes, no)	No data
	Incubation duration (short, medium, **long**, extralong)	Thorbjarnarson (1996)
*Crocodylus suchus*	Nest site (mound, **hole**, termite)	Fergusson (2010)
	Communal nesting (**yes**, no)	Pooley (1977)
	Nest defense (**yes**, no)	Fergusson (2010)
	Nest attendance (**yes**, no)	Fergusson (2010)
	Hatchling attendance (**yes**, no)	Fergusson (2010)
	Attendance lodging (water, burrow, wallow)	No data
	Hatching stimulus (signal calling, vibration)	No data
	Nesting season (wet, **dry**)	Fergusson (2010)
	Clutch frequency (1, 2)	No data
	Clutch size (small, medium, **large**)	Thorbjarnarson (1996)
	Clutch mass (**small,** medium, large)	Thorbjarnarson (1996)
	Mating vocals (none, **roar**, bellow, buzz, moan)	Dinets (2013)
	Wet season hatching (yes, no)	No data
	Incubation duration (short, medium, **long**, extralong)	Thorbjarnarson (1996)
*Mecistops cataphractus*	Nest site (**mound**, hole, termite)	Shirley (2010)
	Communal nesting (yes, **no**)	No report of occurrence
	Nest defense (yes, **no**)	Abercrombie (1978)
	Nest attendance (**yes**, no)	Abercrombie (1978)
	Hatchling attendance (**yes**, no)	Abercrombie (1978)
	Attendance lodging (water, burrow, wallow)	No data
	Hatching stimulus (signal calling, vibration)	No data
	Nesting season (**wet**, dry)	Shirley (2010)
	Clutch frequency (1, 2)	No data
	Clutch size (**small**, medium, large)	Thorbjarnarson (1996)
	Clutch mass (**small**, medium, large)	Thorbjarnarson (1996)
	Mating vocals (none, **roar**, bellow, buzz, moan)	Dinets (2013)
	Wet season hatching (yes, **no**)	Shirley (2010)
	Incubation duration (short, medium, long, **extralong**)	Thorbjarnarson (1996)
*Osteolamus tetraspis*	Nest site (**mound, hole**, termite)	Kofron and Steiner (1994)
	Communal nesting (yes, **no**)	No report of occurrence
	Nest defense (**yes**, no)	Kofron and Steiner (1994)
	Nest attendance (**yes**, no)	Kofron and Steiner (1994)
	Hatchling attendance (yes, no)	No data
	Attendance lodging (water, burrow, wallow)	No data
	Hatching stimulus (signal calling, vibration)	No data
	Nesting season (**wet**, dry)	Kofron and Steiner (1994)
	Clutch frequency (**1**, 2)	Kofron and Steiner (1994)
	Clutch size (**small**, medium, large)	Thorbjarnarson (1996)
	Clutch mass (small, **medium**, large)	Thorbjarnarson (1996)
	Mating vocals (none, roar, bellow, buzz, **moan**)	Dinets (2013)
	Wet season hatching (yes, **no**)	Kofron and Steiner (1994)
	Incubation duration (short, medium, long, **extralong**)	Thorbjarnarson (1996)
*Crocodylus porosus*	Nest site (**mound**, hole, termite)	Webb, Messel, and Magnusson (1977)
	Communal nesting (yes, **no**)	No report of occurrence
	Nest defense (**yes**, no)	Lang (1987)
	Nest attendance (**yes**, no)	Lang (1987)
	Hatchling attendance (**yes**, no)	Webb et al. (1977)
	Attendance lodging (water, burrow, **wallow**)	Webb et al. (1977)
	Hatching stimulus (**signal calling**, vibration)	Webb et al. (1977)
	Nesting season (**wet**, dry)	Webb et al. (1977)
	Clutch frequency (**1**, 2)	Webb et al. (1977)
	Clutch size (small, **medium**, large)	Thorbjarnarson (1996)
	Clutch mass (small, **medium**, large)	Thorbjarnarson (1996)
	Mating vocals (none, **roar**, bellow, buzz, moan)	Grigg and Kirshner (2015)
	Wet season hatching (yes, **no**)	Webb et al. (1977)
	Incubation duration (short, medium, long, **extralong**)	Thorbjarnarson (1996)
*Crocodylus johnstoni*	Nest site (mound, **hole**, termite)	Webb, Manolis, and Buckworth (1983)
	Communal nesting (**yes**, no)	Webb, Buckworth, and Manolis (1983)
	Nest defense (yes, **no**)	Webb, Manolis, et al. (1983)
	Nest attendance (yes, **no**)	Webb, Manolis, et al. (1983)
	Hatchling attendance (**yes**, no)	Webb, Manolis, et al. (1983)
	Attendance lodging (**water**, burrow, wallow)	Webb, Manolis, et al. (1983)
	Hatching stimulus (**signal calling**, vibration)	Webb, Manolis, et al. (1983)
	Nesting season (wet, **dry**)	Webb, Manolis, et al. (1983)
	Clutch frequency (**1**, 2)	Webb, Manolis, et al. (1983)
	Clutch size (**small**, medium, large)	Thorbjarnarson (1996)
	Clutch mass (**small**, medium, large)	Thorbjarnarson (1996)
	Mating vocals (none, **roar**, bellow, buzz, moan)	Dinets (2013)
	Wet season hatching (**yes**, no)	Webb, Manolis, et al. (1983)
	Incubation duration (short, **medium**, long, extralong)	Thorbjarnarson (1996)
*Crocodylus palustris*	Nest site (mound, **hole**, termite)	Da Silva and Lenin (2010)
	Communal nesting (**yes**, no)	No report of occurrence
	Nest defense (yes, **no**)	Grigg and Kirshner (2015)
	Nest attendance (yes, **no**)	Da Silva and Lenin (2010)
	Hatchling attendance (**yes**, no)	Whitaker and Whitaker (1984)
	Attendance lodging (**water**, burrow, wallow)	Da Silva and Lenin (2010)
	Hatching stimulus (**signal calling**, vibration)	Whitaker and Whitaker (1984)
	Nesting season (wet, **dry**)	Lang, Andrews, and Whitaker (1989)
	Clutch frequency (1, **2**)	Da Silva and Lenin (2010)
	Clutch size (**small**, medium, large)	Thorbjarnarson (1996)
	Clutch mass (**small**, medium, large)	Thorbjarnarson (1996)
	Mating vocals (none, **roar**, bellow, buzz, moan)	Dinets (2013)
	Wet season hatching (**yes**, no)	Lang et al. (1989)
	Incubation duration (short, **medium**, long, extralong)	Thorbjarnarson (1996)
*Crocodylus mindorensis*	Nest site (**mound**, hole, termite)	Van Weerd (2010)
	Communal nesting (yes, **no**)	No report of occurrence
	Nest defense (**yes**, no)	Van Weerd (2010)
	Nest attendance (**yes**, no)	Van Weerd (2010)
	Hatchling attendance (yes, no)	No data
	Attendance lodging (water, **burrow**, wallow)	Van Weerd et al. (2006)
	Hatching stimulus (signal calling, vibration)	No data
	Nesting season (wet, **dry**)	Van Weerd (2010)
	Clutch frequency (1, **2**)	Van Weerd (2010)
	Clutch size (small, **medium**, large)	Thorbjarnarson (1996)
	Clutch mass (**small**, medium, large)	Thorbjarnarson (1996)
	Mating vocals (none, roar, **bellow**, buzz, moan)	Dinets (2013)
	Wet season hatching (**yes**, no)	Van Weerd (2010)
	Incubation duration (short, medium, **long**, extralong)	Thorbjarnarson (1996)
*Crocodylus siamensis*	Nest site (**mound**, hole, termite)	Sam et al. (2015)
	Communal nesting (yes, **no**)	No report of occurrence
	Nest defense (**yes**, no)	Sam et al. (2015)
	Nest attendance (**yes**, no)	Sam et al. (2015)
	Hatchling attendance (**yes**, no)	Whitaker (2007)
	Attendance lodging (water, burrow, wallow)	No data
	Hatching stimulus (signal calling, vibration)	No data
	Nesting season (wet, **dry**)	Sam et al. (2015)
	Clutch frequency (**1**, 2)	Sam et al. (2015)
	Clutch size (small, **medium**, large)	Thorbjarnarson (1996)
	Clutch mass (small, **medium**, large)	Thorbjarnarson (1996)
	Mating vocals (none, **roar**, bellow, buzz, moan)	Dinets (2013)
	Wet season hatching (**yes**, no)	Sam et al. (2015)
	Incubation duration (short, **medium**, long, extralong)	Thorbjarnarson (1996)
*Crocodylus novaeguineae*	Nest site (**mound**, hole, termite)	Hall and Johnson (1987)
	Communal nesting (yes, **no**)	No report of occurrence
	Nest defense (**yes**, no)	Hall and Johnson (1987)
	Nest attendance (**yes**, no)	Hall and Johnson (1987)
	Hatchling attendance (**yes**, no)	Hall and Johnson (1987)
	Attendance lodging (water, burrow, **wallow**)	Hall and Johnson (1987)
	Hatching stimulus (signal calling, vibration)	No data
	Nesting season (**wet, dry**)	Hall (1989)
	Clutch frequency (1, 2)	No data
	Clutch size (small, **medium**, large)	Thorbjarnarson (1996)
	Clutch mass (small, **medium**, large)	Thorbjarnarson (1996)
	Mating vocals (none, **roar**, bellow, buzz, moan)	Dinets (2013)
	Wet season hatching (yes, **no**)	Hall and Johnson (1987)
	Incubation duration (short, **medium**, long, extralong)	Thorbjarnarson (1996)
*Tomistoma schlegelii*	Nest site (**mound**, hole, termite)	Bezuijen, Shwedick, Sommerlad, Stevenson, and Steubing (2010)
	Communal nesting (yes, **no**)	No report of occurrence
	Nest defense (**yes**, no)	Grigg and Kirshner (2015)
	Nest attendance (**yes**, no)	Grigg and Kirshner (2015)
	Hatchling attendance (yes, no)	No data
	Attendance lodging (water, burrow, **wallow**)	Stuebing, Sommerlad, and Staniewicz (2015)
	Hatching stimulus (signal calling, vibration)	No data
	Nesting season (wet, **dry**)	Stuebing et al. (2015)
	Clutch frequency (1, 2)	No data
	Clutch size (small, **medium**, large)	Thorbjarnarson (1996)
	Clutch mass (**small**, medium, large)	Thorbjarnarson (1996)
	Mating vocals (**none**, roar, bellow, buzz, moan)	Dinets (2013)
	Wet season hatching (yes, no)	No data
	Incubation duration (short, medium, **long**, extralong)	Thorbjarnarson (1996)
*Gavialis gengeticus*	Nest site (mound, **hole**, termite)	Lang and Kumar (2013)
	Communal nesting (**yes**, no)	Katdare et al. (2011)
	Nest defense (**yes**, no)	Grigg and Kirshner (2015)
	Nest attendance (**yes**, no)	Grigg and Kirshner (2015)
	Hatchling attendance (**yes**, no)	Lang and Kumar (2013)
	Attendance lodging (**water**, burrow, wallow)	Lang and Kumar (2013)
	Hatching stimulus (**signal calling**, vibration)	Lang and Kumar (2013)
	Nesting season (wet, **dry**)	Lang and Kumar (2013)
	Clutch frequency (**1**, 2)	Lang and Kumar (2013)
	Clutch size (small, medium, **large**)	Thorbjarnarson (1996)
	Clutch mass (**small**, medium, large)	Thorbjarnarson (1996)
	Mating vocals (none, roar, bellow, **buzz**, moan)	Dinets (2013)
	Wet season hatching (yes, **no**)	Lang and Kumar (2013)
	Incubation duration (short, medium, **long**, extralong)	Thorbjarnarson (1996)

Note

Bolded character states indicate the character state for the listed species.

## 3 RESULTS

### 3.1 Mating vocalizations

**Figure 1:**
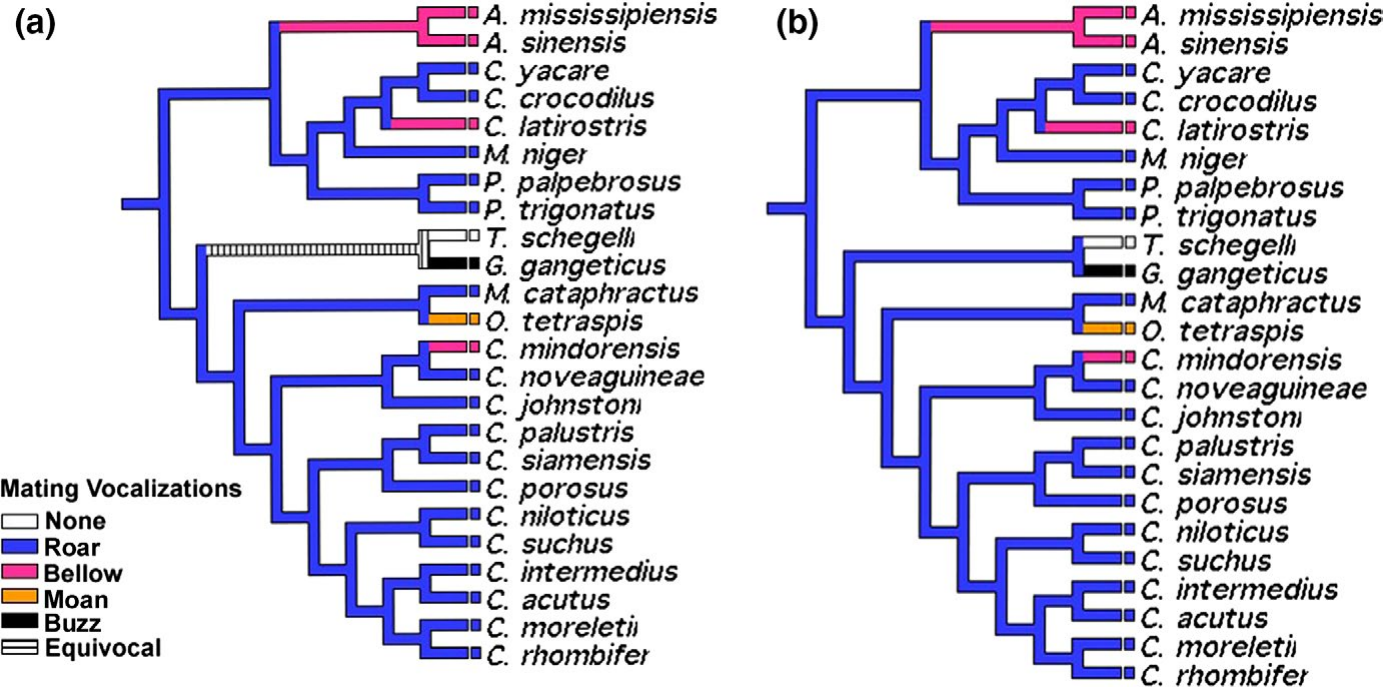
Character optimization of mating vocalizations using accelerated (a) and delayed (b) transformations

Accelerated transformations (AT) and delayed transformations (DT) indicate a mating roar as the ancestral state among crocodilians (Figure 1a,b). This plesiomorphy was followed by the independent and convergent evolution of bellowing vocalizations in *Alligator* as well as *Caiman latirostris,* and *Crocodylus mindorensis* of the Pacific Philippine Islands. Additionally, the independent evolution of no vocalizations, a “buzz,” and “moan” occurred in the monotypic genera *Tomistoma, Gavialis*, and *Osteolaemus*, respectively (Figure 1). Discrepancies among AT and DT result from an algorithmic assignment of the ancestral character state for Gavialidae in which AT recovers an equivocal ancestral state for the family and DT recovers “roar” as the ancestral condition for Gavialidae.

### 3.2 Nesting mode and communal nesting

**Figure 2:**
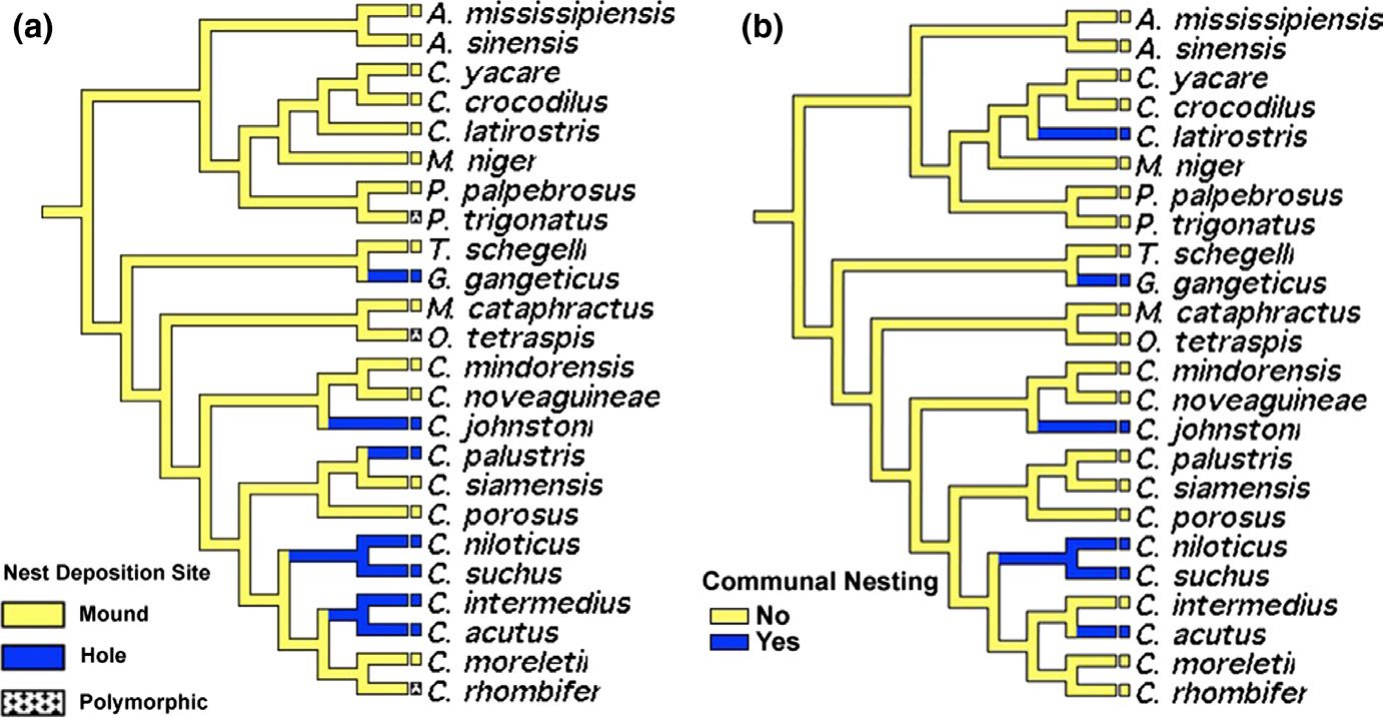
Character optimization of nesting mode (a) and communal nesting (b). Accelerated and delayed transformations recovered unequivocal results for both characters

Both AT and DT suggest mound nesting as the ancestral state among crocodilians, with the independent evolution of hole nesting in New World *Crocodylus *(*C. acutus* and *C. intermedius*) and the following Old World species: *Crocodylus palustris, C. johnstoni, C. suchus, C. niloticus*, and *Gavialis* (Figure 2a,b). The ancestral character state for African and New World *Crocodylus* is the only discrepancy among AT and DT, resulting in either a reversal to mound nesting in *C. moreletii* or the convergence to hole nesting in the rest of African and New World *Crocodylus*, as *C. rhombifer* remains polymorphic for nesting mode. One enigmatic apomorphy is the use of termite mounds (coded as mounds) by *Paleosuchus trigonatus*, an alligatorid. Furthermore, the independent evolution of communal nesting behaviors has occurred five times, within Alligatoridae (*Caiman latirostris*), and in one Australian, both African, and one New World member of the genus *Crocodylus* (*C. johnstoni*, *C. suchus* and *niloticus*, and *C. acutus*, respectively), as well as *Gavialis*. Results were unequivocal among transformation types.

### 3.3 Maternal nest attendance and lodging, nest defense, and hatchling attendance

**Figure 3:**
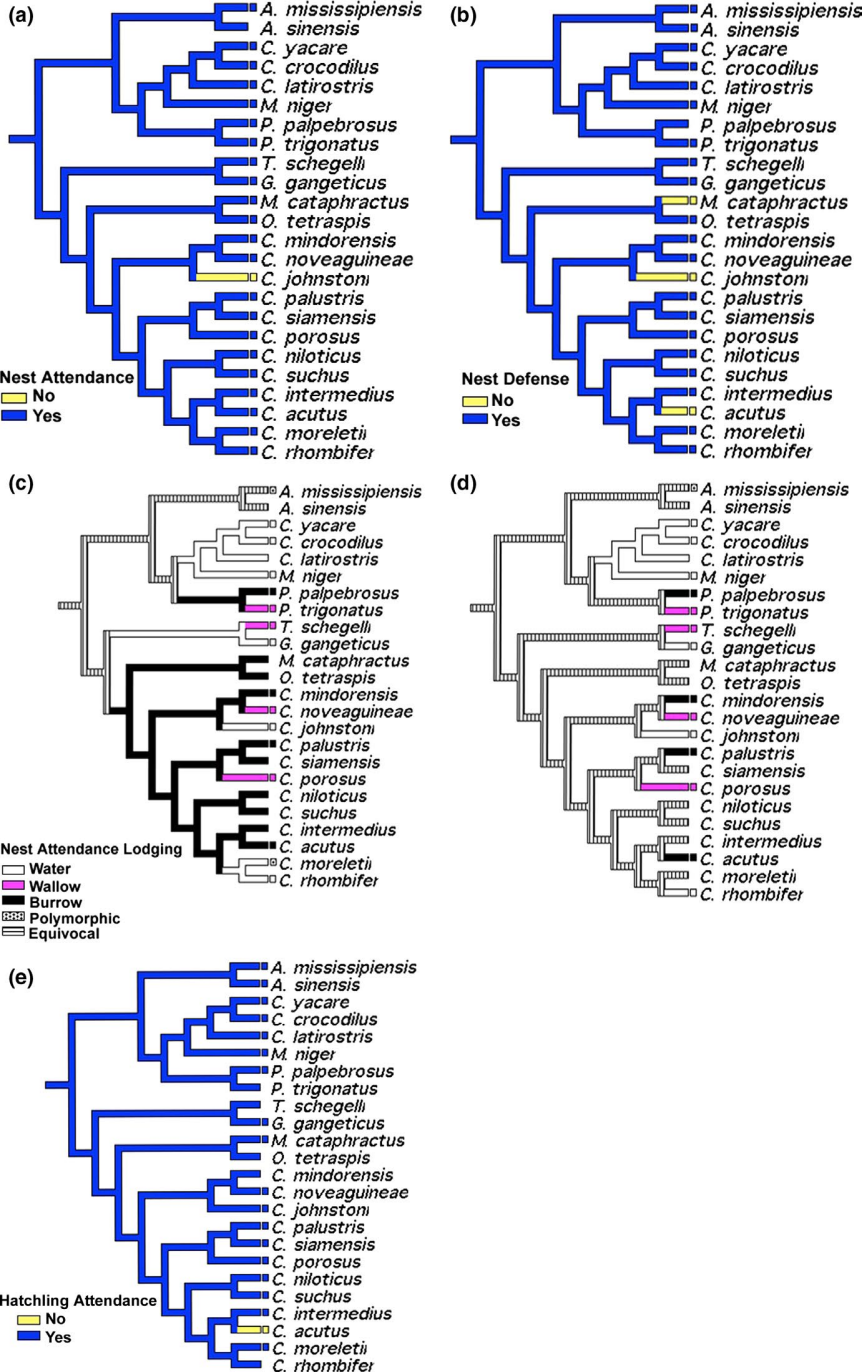
Character optimization of nest attendance (a) and nest defense (b). Accelerated and delayed transformations recovered unequivocal results for both characters. Additionally, character optimization of nest attendance lodging using accelerated (c) and delayed (d) transformations, as well as unequivocal character optimization for hatchling attendance (e)

Every species of crocodilian aside from *Crocodylus johnstoni* exhibits nest attendance, defined here as the maintenance of active positioning of the mother in close proximity to the nest; therefore, its origins may be in an earlier lineage (Figure 3a–e). Nest defense, or the active aggressive defending of the nest by the mother, is also characteristic of most crocodilians and their hypothetical ancestors. This behavior has apparently been lost independently in *C. johnstoni*,* C. acutus*, and in the monotypic *Mecistops*. The lodging by which nests are attended, however, is more heterogeneous. Both AT and DT indicate nest attendance in nearby water and/or use of burrows as the ancestral state for Crocodylia, with convergences to wallow use (cleared swath of nearby land) in all three families. Both transformation types recover water attendance for *Caiman* and *Melanosuchus*. Accelerated transformations indicate the use of burrows (underground hole in the bank) as the ancestral condition for Crocodylidae with reversals to water or wallow attendance at terminal nodes. Delayed transformations, however, recover equivocal character states between water attendance and burrow use for all internal nodes aside from those basal to terminal *Caiman* and *Melanosuchus*. Additionally, hatchling attendance evolution was unequivocal with only *Crocodylus acutus* not being predominantly attentive to hatchling pods, after nest opening.

### 3.4 Clutch frequency

**Figure 4:**
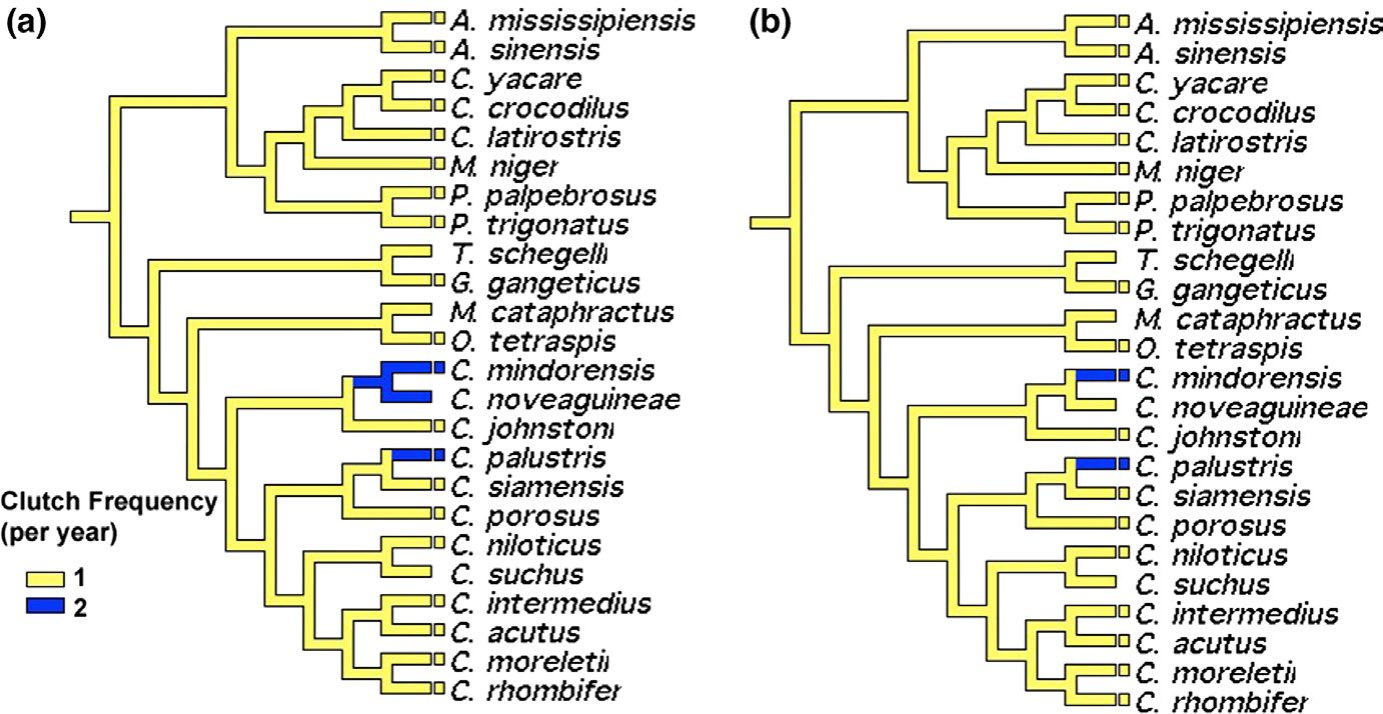
Character optimization of clutch frequency using accelerated (a) and delayed (b) transformations

The ability to produce multiple clutches within the same nesting season has only been documented in two species: *Crocodylus mindorensis* and *C. palustris* (Figure 4a,b). Naturally, all analyses indicate one clutch per nesting season as the ancestral condition for Crocodylia, with discrepancies between transformation types resulting from missing data for *C. novaeguineae*.

### 3.5 Seasonal timing of hatching, hatching stimulus, and nesting season

**Figure 5:**
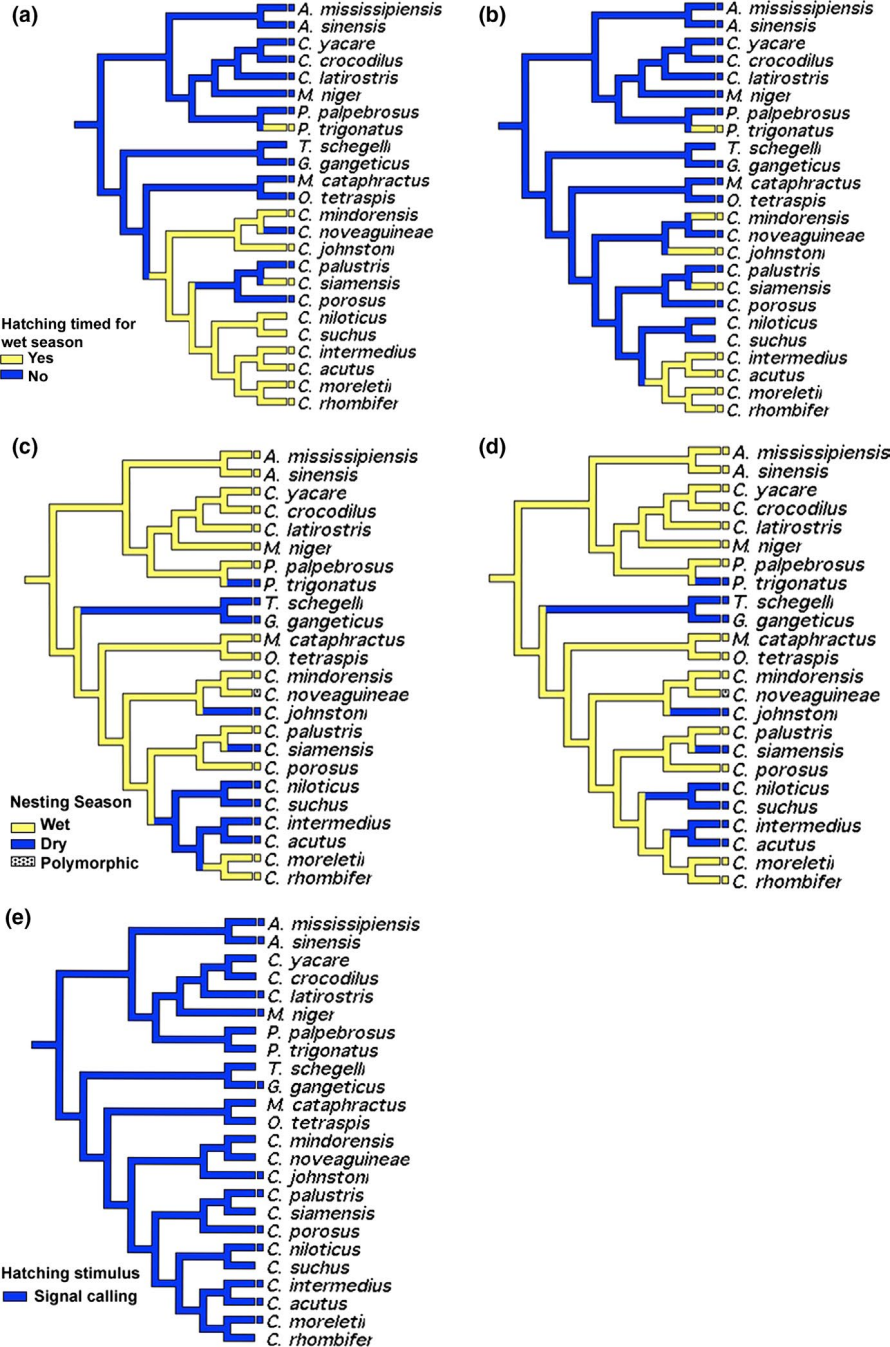
Character optimization of hatching timed for wet season using accelerated (a) and delayed (b) transformations; nesting season using accelerated (c) and delayed (d) transformations, and hatching stimulus (e). Accelerated and delayed transformations recovered unequivocal results for this character

Timing hatching with the onset of the wet season theoretically provides better cover and resource access to emerging hatchlings (e.g., Doody, Georges, & Young, 2001); however, as hypothesized by AT, this trait has evolved only within the Crocodylidae with reversals in *C. novaeguineae*,* C. palustris*, and *C. porosus* (Figure 5a–e). Additionally, *P. trigonatus* shows independent convergence on this reproductive timing. Delayed transformations indicate that hatching timed for the wet season only characterizes New World *Crocodylus* with three additional convergences within the genus as well as *P. trigonatus*. It is worth noting that eggs of *Alligator mississippiensis* hatch with the onset of the wettest months at the northernmost periphery of the distribution (Wilkinson, 1983), so variation in this character may exist in wide‐ranging taxa.

The evolution of hatching stimuli was uninformative after a literature review recovered no alternative character states to signal calling in all species for which information was available. Nesting season, however, presents more heterogeneous character states. Transformation types agree on the convergent evolution of dry season nesting in *Gavialidae*, *C. johnstoni,* and *Alligator sinensis*, as well as the alligatorid *P. trigonatus*. Additionally, discrepancies between transformation types resulted in equivocal data for the ancestral condition of African and New World *Crocodylus*, with sister taxa *Crocodylus morletii* and *C. rhombifer* nesting during the wet season.

### 3.6 Incubation period, relative clutch mass, and clutch size

**Figure 6:**
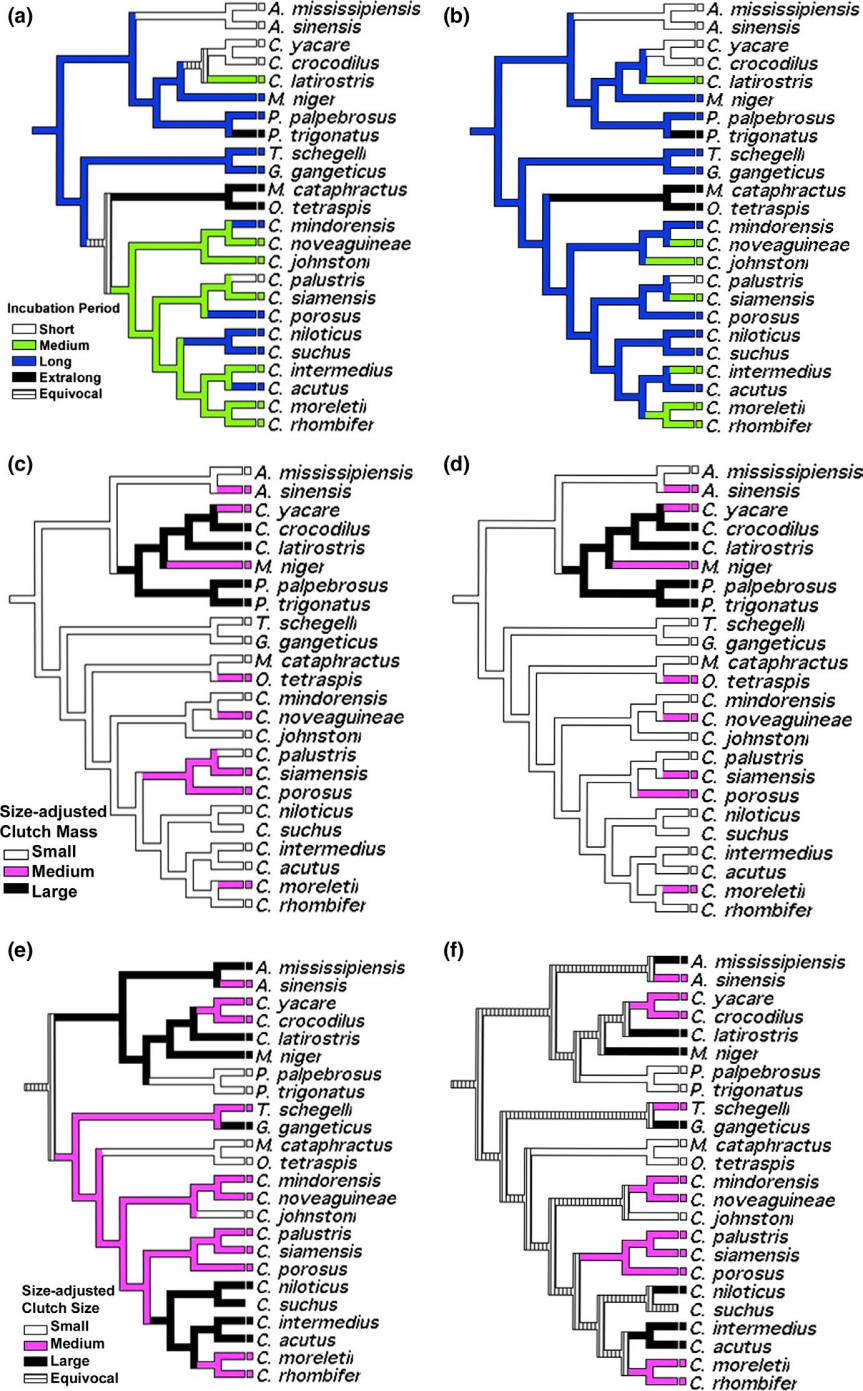
Character optimization of incubation duration using accelerated (a) and delayed (b) transformations; size‐adjusted clutch mass using accelerated (c) and delayed (d) transformations, and size‐adjusted clutch size using accelerated (e) and delayed (f) transformations

Accelerated transformations revealed high variation in duration of the incubation period (Figure 6a–f). The hypothetical ancestor for Crocodylia was characterized by a long incubation period (85–90 days), a character state exhibited by basal Alligatoridae as well as Gavialidae. While data for the ancestral condition for Crocodylidae are equivocal, only the two monotypic genera (*Mecistops* and *Osteolameus*) exhibit extralong incubation periods (~100 days). The genus *Crocodylus* is characterized by medium incubation durations (75–80 days) with reversals to long incubation periods in *C. mindorensis*,* C. porosus*,* C. suchus*,* C. niloticus,* and *C. acutus* and short incubation periods (65–70 days) in *C. palustris*. Members of Alligatoridae exhibit all character states with convergence to short incubation times in the genus *Alligator*, *Caiman yacare*, and *C. crocodilus*. The rest of Alligatoridae exhibited medium, long, and extralong incubation periods that lack evolutionary signal. Delayed transformations recovered a long incubation period as the ancestral state for the genus *Crocodylus*, with six convergences on shorter incubation periods.

The evolution of (size‐adjusted) clutch mass is congruent between transformation types. The ancestor to crocodilians was characterized by a small clutch mass (3.7%–5.7% female mass) with numerous convergences at terminal nodes to medium‐sized clutch masses (6.5%–8.5%). Large clutch mass (13.5%–16.4%) evolved in the hypothetical ancestor for caimans including the genus *Paleosuchus*. Conversely, despite an equivocal character state for the hypothetical ancestor, the (size‐adjusted) clutch size analyses suggest medium‐sized (23.4–31.8 eggs) or large clutches (35.4–47.6 eggs) as the plesiomorphic state. Medium clutch sizes characterize Gavialidae and Crocodylidae, with convergence to large clutch sizes in *Gavialis* and New World *Crocodylus*, with a reversal back to medium clutch sizes in *C. morletii* and *C. rhombifer*. The terminal *Crocodylus* with medium clutch masses also have medium clutch sizes, while all taxa and ancestors with large clutch masses are characterized by small clutch sizes (12.3–19.5 eggs) with the exception of *C. latirostris* and *Melanosuchus niger*.

## 4 DISCUSSION

We used a contemporary phylogenetic hypothesis and an improved knowledge of species‐specific reproductive behaviors and life‐history strategies to infer reproductive trait evolution in crocodiles. By tracking traits through crocodilian evolution, our analysis provided a first‐ever complete look at when those traits evolved, facilitating informative speculation on why they evolved. Our analysis revealed that the ancestral crocodilian was a mound nester, with 4–5 transformations to hole nesting; habitat may have driven mode of nesting (mound vs. hole), with mound nesters occupying mainly swamps and marshes and hole nesters inhabiting large rivers and lakes, at least during the nesting season. Hole nesters were more likely to nest communally, but this association may be biased by scale. Although there were exceptions, mound nesters nested during the wet season and holes nesters during the dry season; this trait was relatively conserved, however. Nest attendance and defense were nearly ubiquitous, and thus, our analysis exhibited phylogenetic constraint for these characters, but attendance lodging was diverse among species, showing multiple reversals between water and burrows. Finally, about two‐thirds of species timed their *nesting* with the wet season, while the other third timed their *hatching* with the onset of the wet season. Collectively, our analysis provides a framework for understanding reproductive trait evolution in crocodilians.

All crocodilians vocalize, but there is some variation in the types of calls they make (reviewed in Vergne, Pritz, & Mathevon, 2009; Grigg & Kirshner, 2015). In the present study, we analyzed mating calls, which were overwhelmingly described as bellows or roars in all but three species (Figure 1). Although it makes distress calls (Bonke, Whitaker, Roedder, & Boehme, 2015), *T. schlegelli* is not known to make mating calls. Its sister species, *Gavialis gangeticus*, makes a loud “buzz,” while the call of *Osteolamus tetraspis* has been described as a “moan.” Our analyses thus suggest that at least the general sounds audible to human ears are conserved among crocodilians, with the recent loss or modification of calls in the three abovementioned species. However, the contexts of these calls, including habitat, sex, and season, along with associated behavioral traits (e.g., head slapping, back vibrations—see Vergne et al., 2009 and references therein) can be species‐specific and thus the evolution of call repertoires in crocodilians requires further observations and comparative analysis. Ultimately, a comparative study using sonogram signatures is required to truly understand vocalization evolution in crocodilians.

Most crocodilians excavate and backfill a nest either in a large mound constructed of mud and/or vegetation, in the ground (Campbell, 1972; Greer, 1970; Thorbjarnarson, 1996). After reviewing nesting mode in crocodilians, Greer (1970) hypothesized that the “common ancestor of the gavialids and crocodylines, and therefore of all living crocodilians, was probably a hole nester.” Grigg and Kirshner (2015) countered that the ancestor was likely a mound nester, based on newer phylogenetic hypotheses and additional data on mode of nesting. Our analysis suggests that the ancestral crocodilian was indeed a mound nester, with 4–5 independent transitions to hole nesting, depending on transformation type (Figure 2; 3–4 within Crocodylidae and one within Gavialidae). To be fair, crocodilian relationships were poorly known at the time of Greer's contention; for example, *T. schlegelli*, which is now recognized as the sister to *G. gangeticus* (and part of Gavialidae), was at the time considered to be a monotypic species within the Crocodylidae, and some of his species pairs are no longer recognized as such (e.g., *novaeguineae‐porosus*,* niloticus‐palustris*). Greer (1970) noted apparent support for his hypothesis in other reptiles: Hole nesting is common in turtles, lizards, and the tuatara. However, we now have a more accurate understanding of the relationships among turtles, lepidosaurs, birds, and crocodilians. Among archosaurs, mound nesting occurs in megapode birds and possibly occurred in an extinct crocodilian (*Diplocynodon darwini*, Hastings & Hellmund, 2015). Dinosaurs very likely exhibited a diversity of nesting types given the length of their tenure; however, it is very difficult to determine (from fossils) if their eggs were laid on the ground, buried in the ground, or in a mound of vegetation (Carpenter, 1999). Although vegetation from any dinosaur mound nests would have long rotted and disappeared, leaving only clutches of eggs in rocks, there are hypotheses with some circumstantial evidence for mound nesting in *Oviraptor* and other species (Coombs, 1989; Horner & Makela, 1979; Sabath, 1991; Carpenter, 1999). There is, however, good evidence for hole nesting in dinosaur fossils: Both multilayered and erect eggs must have been at least partially buried in order to keep their positions (Cousin, Breton, Fournier, & Watte, 1994; Carpenter, 1999). Hole nesting is nearly ubiquitous in turtles, with only two species exhibiting mound nesting (*Manouria* spp.). Collectively, then, early crocodilians may have evolved mound nesting from a hole‐nesting lineage, but the uncertainty of the distribution of nesting types among fossil reptiles, along with ambiguity on the phylogenetic position among turtles, archosaurs, and lepidosaurs (Field et al., 2014), prevents confident assignment.

Are there any clues to why mode of nesting evolves in extant crocodilians? Greer (1970) found no clear association between mode of nesting and the general ecology or behavior of species, suggesting instead that mode of nesting is an ancient trait in crocodilians. In contrast, Neill (1971) and Campbell (1972) contended that the nesting mode of crocodilians was related to habitat, with mound nesting in marshy environments and hole nesting on banks. Two opposing camps thus attempted to explain mode of nesting in crocodilians; the phylogenetic camp (Schmidt, 1924; Greer, 1970; but see also Greer, 1970) and the ecological camp (Wermuth, 1953; Campbell, 1972; Neill, 1971; Ouboter & Nanhoe, 1987; Thorbjarnarson, 1996). Our analysis demonstrated repeated convergence to mound nesting and, thereby, could not falsify an association between mode of nesting and ecological factors such as habitat type. All hole nesters seemingly include rivers in their breeding habitat types, even if the species occupies a diversity of habitats; conversely, all mound nesters seemingly include marshes or swamps in their breeding habitat types (as per Campbell, 1972). While the evolution of nest site in association with lentic or lotic habitats was not directly tested here, our analysis provides reasonable evidence for habitat type driving mode of nesting. However, tracking mode of nesting through the lineage also reveals conservation of nesting mode: The ancestral character state of mound nesting has persisted in most species, with hole nesting appearing much more recently. Hole nesting has also persisted for some time within a group comprising four species of crocodylids, however (*C. niloticus*,* C. suchus*, *C. intermedius*, and *C. acutus*). Unfortunately, we cannot readily and reliably retrieve habitat types for ancestral (fossil) crocodilians to confirm that habitat has driven mode of nesting in crocodilians. However, based on our analyses it is unequivocal that mound nesting is the plesiomorphic and conserved condition and that hole nesting is a derived convergent behavior. If nesting type can be confidently tied to nest habitat, then at minimum mound nesting appears conserved because where crocodilians live is a conserved trait.

The apparent presence of both mound and hole nesting in some species generates the hypothesis that mode of nesting reflects (individual) behavioral plasticity. This is further supported by intraspecific hole nesting, mound nesting, and intermediates (low, poorly differentiated mounds) in *C. acutus* in Florida and Belize (Campbell, 1972; Platt & Thorbjarnarson, 2000, respectively) and *A. mississippiensis* in Alabama (Grajal‐Puche, Kearley, Bravo, & Murray, 2018). However, the co‐existence of these two modes of nesting is not sufficient evidence for behavioral plasticity—individuals differing in nesting mode may reflect behavioral polymorphisms. Unequivocal demonstration of behavioral plasticity in mode of nesting would require experiments manipulating nesting (micro) habitat types in outdoor enclosures or transplant experiments moving individuals from one nesting habitat type to another.

At first glance, our analysis revealed an association between communal nesting and nesting mode: Hole nesters tend to nest communally (4 of 6 species = 75%), while only two of 18 species of mound nesters are known to nest communally (*C. palustris* and *C. intermedius*, Figure 2b). However, these differences may be due to a bias of scale. Communal nesting, defined as the deposition of eggs or young with those of conspecifics (Doody, Keogh, & Freedberg, 2009), can be conspicuous in hole nesters, which tend to aggregate their nests on sand banks and may not defend them (e.g., *C. johnstoni*), while mound nesters are in more direct attendance which would prevent multiple mothers from laying in the same mound. Accordingly, multiple clutches are rarely found within one nest [but see Larriera (2002) for an exception in *C. latirostris* or P. Wilkinson (personal communication) for an exception in *A. mississippiensis*). However, communal nesting at the larger scale of a cluster of mound nests in a given area of a swamp or marsh can occur and may be more common than appreciated. It is quite possible that clumping of mound nests at such a larger scale has gone undetected in many species. Regardless, communal nesting occurs in other archosaurs (definitely in birds and probably in dinosaurs; Carpenter, Hirsch, & Horner, 1994; Carpenter, 1999) and thus is an ancient trait that can be explained by social behaviors, limited nest sites, predation guarding efficiency, or some combination of these (Doody et al., 2009).

Our analyses of nest attendance exhibited phylogenetic constraint (Figure's 3a–c). However, attendance lodging was diverse among species, showing multiple reversals between water and burrows under one transformation type, along with recent origins of wallow use (Figure 3d). Thus, the behavior of utilizing a particular microhabitat appeared particularly evolutionarily labile. There appeared to be no relationship between attendance lodging and mode of nesting. Nest attendance interpreted from the fossil crocodile *Diplocynodon darwini* was somewhat expected, because of the species' nested phylogenetic position within the crown group Crocodylia (Hastings & Hellmund, 2015). The discovery of nest attendance in three species of theropod dinosaurs from the late Cretaceous (Norell et al., 1995; Dong & Currie, 1996; Varricchio, Jackson, Borkowski, & Horner, 1997) demonstrates that nest attendance is an ancient trait that extended into the nonavian theropod lineage (Hechenleitner, Grellet‐Tinner, & Fiorelli, 2015).

Fifteen of the 24 species in our analysis nest during the wet season (Figure 5c,d). There appeared to be an association between nesting season (wet vs. dry) and mode of nesting, with hole nesters tending to nest during the dry season and mound nesters nesting during the wet season. However, there were exceptions to this association (e.g., mound nesting during the dry season in *Mecistops cataphractus*, *O. tetraspis,* and *Crocodylus siamensis*, and hole nesting during the wet season in *C. palustris*). Interestingly, while the ancestral state was wet season nesting with six independent origins of dry season nesting, there were no reversals, suggesting that nesting during a distinct season is relatively conserved. Relatedly, eight of 24 species exhibited hatching that was timed with the onset of the wet season (Figure 5a,b). As with nesting season, reversals apparently did not occur (Figure 5c,d). Thus, collectively, two‐thirds of crocodilian species time their *nesting* with the wet season and one‐third times their *hatching* to the wet season, and we hypothesize that benefits accrue to eggs and hatchings, respectively. A caveat here is that the geographic distribution (or part thereof) of some species does not include a distinct wet/dry season. For example, *A. mississippiensis* is abundant in Louisiana, which has very little seasonal variation in rainfall (Keim & Faiers, 1996). In other words, in some cases timing of nesting or hatching in crocodilians may not be an adaptation to synchronize some part of the life cycle to the wet season. Of course, alternative zeitgebers may be driving the timing of these events.

Clutch frequency is remarkably conserved in crocodilians; only two species are known to lay two clutches per year, both of which are tropical (Figure 4; *C. palustris*,* C. mindorensis*). Not surprisingly, our finding is consistent with higher reproductive frequency in crocodylids compared to alligatorids (Thorbjarnarson, 1996). Relative clutch mass appeared to be ancestrally small in crocodilians, with 6–7 independent transitions to mainly medium‐sized clutch masses, but with one transition to large clutch mass in the Caimaninae, followed by a reversal to medium in *C. yacare* and *M. niger* (Figure 6c,d). Relative clutch mass was higher in alligatorids than in gavialids and crocodylids, supporting the findings of Thorbjarnarson (1996). Size‐adjusted clutch size was highly variable among species, with 11 medium‐sized, six large, and five small clutch sizes (Figure 6e,f). Although there were some clear transitions (e.g., medium to small clutch size in *C. johnstoni*; large to small clutch sizes in *Paleosuchus*), the lack of a clear ancestral character state resulted in ambiguity (Figure 6e,f). Moreover, we did not consider egg size and the potential for a tradeoff between egg size and clutch size. However, quantitative analyses have found no such tradeoff among crocodilian species (Platt, Rainwater, Thorbjarnarson, & McMurry, 2008; Thorbjarnarson, 1996; Thorbjarnarson & Hernández, 1993).

**Figure 7:**
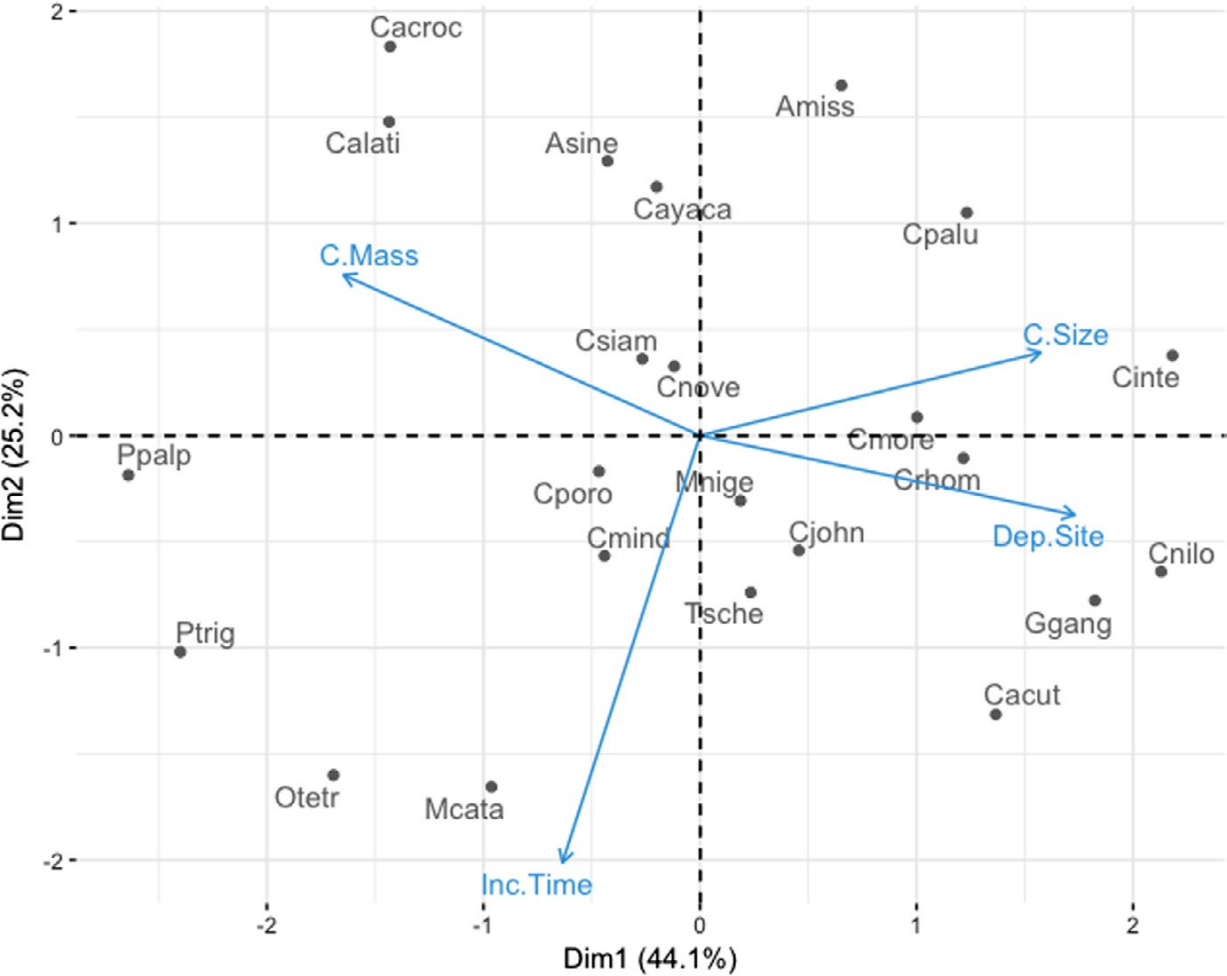
Principal components analysis by which clutch mass, clutch size, incubation period, and deposition site (categorical) arrange species in ordination space. This elucidates that hole‐nesting crocodilians had larger clutch sizes than mound nesters

Our results indicate that the hypothetical ancestor for Crocodylia was a mound nester that exhibited nest attendance and active defense from nearby water or burrows. Nesting occurred in the wet season and mating “roars” were used as social copulatory strategies. This animal was likely not a communal nester and deposited one reproductive output annually. Additionally, this species had long egg incubation periods with an average or large number of relatively small eggs. Our results also suggest that the evolution of clutch mass, clutch size, incubation period, and nest site are correlated, with lineages commonly exhibiting small clutch sizes and larger clutch masses, mound nesting, and short incubation periods. An ad hoc principal components analysis was performed using species as the response variables by which clutch mass, clutch size, incubation period (all on a continuous distribution), and nest site (categorical) arranged species in ordination space to visualize the relative influence of these variables on one another (Figure 7). Hole nesting species had larger clutch sizes than mound nesting species. Incubation period appears to be independent of any other variable, a result that is supported by the strong relationship between incubation temperature and incubation period (Ewert, 1979, 1985). The relationship between clutch morphometrics and nest type was predicted by Seymour and Ackerman (1980) from the perspective of respiratory constraints on developing embryos. They posited that decomposing organic material around a clutch of eggs imposes more extreme gas tensions than soil or sand, through which gas readily diffuses and nest gas tensions, varying as a function of nesting material, provide constraints on clutch size. We corroborate this finding using the clutch morphometrics and nest sites of all extant crocodilian species.

Here, we present data that diagnose the evolution of nesting ecology within the highly successful Crocodylia. Results diagnose a combination of phylogenetic constraint and convergence, yielding a clade characterized by high evolvability (Brown, 2013), a potential contributor to the success of the lineage over geologic time. In the face of contemporary threats such as climate change, high evolvability of traits directly involved in offspring success may serve well in maintaining the success of the lineage, as it did during previous periods of climatic change. Despite advancements in our understanding of the evolution of nesting ecology and behavior in crocodilians (recent and herein), many questions remain. First, reproductive behavior is still understudied in crocodilians, owed at least in part to their nocturnality, their secretive habits in an aquatic medium, and the difficulties imposed by their large size relative to humans. Second, we have a poor understanding of geographic or other interpopulation variation in reproductive traits within species. For example, we lack sonogram signatures across populations and species; these are needed to fully understand vocalization evolution in the group. Finally, a more careful and robust classification of relative habitat use, in tandem with transplant or common garden experiments, could help clarify the role of habitat in the evolution of reproductive behaviors (e.g., mode of nesting) in this ancient lineage.

## CONFLICT OF INTEREST

We have no competing interests to report.

## AUTHOR CONTRIBUTIONS

CMM conceived and designed the study; acquired, analyzed, and interpreted the data. JSD conceived and designed the study, and acquired and interpreted the data. BIC conceived and designed the study, and analyzed and interpreted the data.

## Data Availability

Raw data used in character optimizations are provided as a table within this manuscript.
